# Outcomes and Hospital Service Use Among Patients With COPD in a Nurse- and Allied Health–Led Clinic

**DOI:** 10.1001/jamahealthforum.2024.1575

**Published:** 2024-07-05

**Authors:** Kailu Wang, Shi Zhao, Susan Zi-May Yau, Yuchen Wei, Yim-Chu Li, Ryan Wai-Ching Orr, Ivan Hin-Lai Lam, Yushan Wu, Eliza Lai-Yi Wong, Chi-Tim Hung, Eng-Kiong Yeoh

**Affiliations:** 1 Centre for Health Systems and Policy Research, JC School of Public Health and Primary Care, Faculty of Medicine, The Chinese University of Hong Kong, Hong Kong, China; 2 School of Public Health, Tianjin Medical University, Tianjin, China; 3 Department of Family Medicine and General Out-patient Clinics, Kowloon Central Cluster, Hospital Authority, Hong Kong, China; 4 Faculty of Medicine, The Chinese University of Hong Kong, Hong Kong, China; 5 Li Ka Shing Faculty of Medicine, The University of Hong Kong, Hong Kong, China

## Abstract

**Importance:**

Multidisciplinary disease management efforts enable the improvement in lung function among patients with chronic obstructive pulmonary disease (COPD), but there is little evidence of its association with risks of adverse health outcomes and health care service use.

**Objective:**

To examine the association between the use of a nurse- and allied health–led primary care clinic for respiratory patients, namely the Nurse and Allied Health Clinic–Respiratory Care (NAHC-Respiratory), and their risks of mortality and morbidity and health care service use.

**Design, Setting, and Participants:**

This territory-wide, population-based, propensity-matched, retrospective cohort study used data from the electronic health records of all patients who used public health care services in Hong Kong, China, from January 1, 2010, to December 31, 2019. All patients with COPD treated in public outpatient clinics between January 1, 2010, and December 31, 2014, were included. Patients who attended NAHC-Respiratory and usual care only were propensity score–matched at a 1:2 ratio. Data analyses were conducted between August 2023 and April 2024.

**Exposure:**

Attendance at NAHC-Respiratory.

**Main Outcomes and Measures:**

All-cause and cause-specific mortality, incidence of COPD complications, and use of emergency department and inpatient services until the end of 2019 were compared between the NAHC-Respiratory and usual care participants using Cox proportional hazard regression, Poisson regression, and log-link gamma regression models after matching.

**Results:**

This study included 9048 eligible patients after matching, including 3093 in the exposure group (2814 [91.0%] men; mean [SD] age, 69.8 [9.5] years) and 5955 in the reference group (5431 [91.2%] men; mean [SD] age, 69.5 [11.7] years). Compared with patients in the usual care–only group (reference), patients in the exposure group had lower risks of all-cause mortality (hazard ratio [HR], 0.84; 95% CI, 0.78-0.90) as well as pneumonia-caused (HR, 0.85; 95% CI, 0.74-0.97), respiratory-caused (HR, 0.86; 95% CI, 0.77-0.96), and cardiovascular-caused (HR, 0.74; 95% CI, 0.59-0.93) mortality. Exposure was associated with reduced rates of emergency department visits (incidence rate ratio [IRR], 0.92; 95% CI, 0.86-0.98) and hospitalization through emergency department (IRR, 0.89; 95% CI, 0.83-0.95).

**Conclusions:**

In this cohort study, the use of a nurse- and allied health–led clinic in primary care settings was associated with reduced risks of mortality and use of hospital services among patients with COPD. These findings emphasize the important role of health care workers other than physicians in disease management in the primary care setting. The NAHC-Respiratory model and service components can be used to help improve primary care programs to benefit more patients with COPD.

## Key Points

**Question:**

Is a multidisciplinary nurse- and allied health–led respiratory primary care clinic associated with a reduced risk of mortality and use of hospital care services among patients with chronic obstructive pulmonary disease (COPD)?

**Findings:**

In this cohort study of 9048 patients with COPD, those who attended the respiratory primary care clinic had a 16% reduction in the risk of all-cause mortality, an 8% reduction in emergency department visits, and lower use of inpatient services in general.

**Meaning:**

The findings suggested that this nurse- and allied health-led clinic in the primary care setting was associated with improved health outcomes and reduced hospital service use among patients with COPD.

## Teaser

This cohort study examines whether a nurse- and allied health–led primary care clinic was associated with improved health outcomes and health care service use among patients with chronic obstructive pulmonary disease (COPD) in Hong Kong.

## Introduction

Chronic obstructive pulmonary disease (COPD) is a group of chronic lung diseases that cause obstructed airflow of the lungs. It is the third leading cause of death globally and poses a substantial disease burden to the global population^1,2^ in addition to contributing to great economic burden, particularly in North America and East Asia.^3^ Smoking, ambient air pollution, and occupational exposure to particulate matter are major factors contributing to the incidence of COPD.^1^ It is associated with subsequent respiratory infections, including pneumonia,^4,5^ respiratory failure,^6^ cardiovascular disease,^7^ gastrointestinal conditions,^8,9,10^ mental disorders,^11^ and other physical disorders in multiple systems,^11^ which could lead to poorer health outcomes, including an increased risk of death.^12,13^

While COPD cannot be cured and could gradually deteriorate, effective disease management interventions are able to improve the prognosis for these patients. Self-management interventions, pulmonary rehabilitation, and other primary care interventions can be used to improve the well-being of patients with COPD,^1,14,15^ and better well-being has been found to be associated with lower risk of COPD exacerbation, hospital admission, and death. To improve health outcomes, services that integrate these interventions have been designed and implemented to enhance COPD management. Previous studies have reported the availability of such integrated COPD management services in Australia,^16^ Canada,^17^ and the Netherlands.^18^ These services involving collaboration and coordination between general physicians, respiratory specialists, allied health professionals, and other therapists have shown improved attendance rates for pulmonary rehabilitation and smoking cessation,^16^ lung function,^17^ and quality of life^17,18^ and reduced number of exacerbations.^17,18^ However, these studies looked at a relatively smaller volume of patients (n <200 in each study) with only 1 year or less of follow-up, and they did not report mortality, morbidity outcomes, or use of different health care services, which are worth investigating to further understand the impact of such services over a longer period.

In Hong Kong, patients with COPD receive care for their pulmonary conditions predominantly in the public health care system. To enhance COPD management in primary care settings, the multidisciplinary Nurse and Allied Health Clinic–Respiratory Care (NAHC-Respiratory) was established in primary care clinics of the public sector in 2009.^19^ While a few analyses have shown that patients with COPD in this program had improvement in their lung function and quality of life during 6- and 12-month follow-up,^20,21^ it is uncertain whether there is an associated lowered mortality and morbidity rate and whether health benefits of the program can be sustained beyond 12 months. Moreover, it is important to find out whether this primary care intervention can save precious health care resources in the secondary and tertiary care settings. Therefore, this study aimed to examine the association between attending NAHC-Respiratory and risk of mortality, incidence of comorbidity and complications, and health care service use during a more than 6-year follow-up period.

## Methods

### NAHC-Respiratory Interventions

This cohort study is based on NAHC-Respiratory, one of the 6 programs under NAHC, which identifies and monitors ambulatory adult patients with COPD in Hong Kong, China. The other 5 programs target mental wellness, wound care, fall prevention, continence care, and medication management. The NAHC-Respiratory program was established and institutionalized in the public sector family medicine and general outpatient clinics and primarily involves nurses, physiotherapists, and occupational therapists. Physicians, nurses, and allied health professionals who provide public primary and secondary outpatient care services can refer ambulatory patients with COPD (ie, treated on an outpatient basis) and those with a high risk of developing COPD (eg, chronic smokers), who can only be referred by physicians, to the NAHC-Respiratory program based on their clinical assessments, and the referred patients may join the program voluntarily. In the NAHC-Respiratory program, health care professionals assess the patient’s spirometry (with and without bronchodilator reversibility test); assess their respiratory symptoms using instruments including the COPD Assessment Test, modified Medical Research Council Dyspnea Scale, and 6-minute walk test; examine their previous exacerbation history; and refer them to different services as appropriate. The services include 3 categories: (1) health education for patients without COPD (ratio of forced expiratory volume in 1 second to forced vital capacity [FEV_1_:FVC] ≥0.7) but at high risk (not included in this study as it was difficult to identify a comparison group), including smoking cessation consultation and redesign of physical exercise and lifestyle; (2) chronic disease self-management for all patients with COPD, including all services in the first category and COPD management consultation on their symptoms, breathing, use of medications, and functional ability; and (3) brief pulmonary rehabilitation program for patients with COPD with functional impairment, including all services in the second category and pulmonary rehabilitation, formulation of an individualized action plan, and education on crisis management and prevention of acute exacerbations.^22^ Patients’ smoking status, vaccination status, and functional ability are assessed during follow-up under the program. Patients are deemed to have completed the program if they have attended at least 75% of the education sessions. They are discharged with possible telephone follow-up and/or community services if they have normal lung function or completed the necessary 6- and 12-month assessments after program intake if they have a clinical diagnosis of COPD. Meanwhile, the program participants are also treated by physicians in outpatient clinics as usual care.

### Usual Care

Regardless of whether they attend the NAHC-Respiratory program, patients with COPD are followed up in the outpatient setting, including family medicine clinics, general outpatient clinics, and specialist outpatient clinics. They may attend these clinics every 3 to 6 months to review their conditions, participate in clinical investigations, and have medications prescribed or adjusted as needed.

### Data Source and Study Sample

Data used in this study were obtained from the Hospital Authority Data Collaboration Lab (HADCL), which is an official health data sharing platform in the Hospital Authority of Hong Kong that consists of all health records of individuals who used services in public hospitals and clinics. The datasets include individual-based patient demographics, disease diagnosis, procedures conducted, medication prescriptions, clinical investigation results, and health care service utilization records. Mortality data were obtained from territory-wide death records maintained by the government through HADCL. This study has been approved by the Survey and Behavioral Research Ethics Committee of the Chinese University of Hong Kong (SBRE-22-0386), and patient consent to participate was waived, as only secondary anonymized data were used. This study was reported according to the Strengthening the Reporting of Observational Studies in Epidemiology (STROBE) reporting guideline.

Considering the referral criteria for the NAHC-Respiratory program, this study includes patients with clinically diagnosed COPD or with a post-bronchodilator FEV_1_:FVC ratio below 0.7 and who were treated in the outpatient setting between January 1, 2010, and December 31, 2014, without severe COPD-related complications at baseline. Patients’ COPD diagnoses were identified based on *International Statistical Classification of Diseases, Tenth Revision, Clinical Modification* (*ICD-10-CM*), and *International Classification of Primary Care, 2nd edition* (*ICPC-2*) codes, as listed in eTable 1 in Supplement 1. Patients with previous diagnoses of common complications of COPD at baseline were excluded, including any pneumonia, respiratory failure, cancers, pulmonary heart disease, pneumothorax, anemia, polycythemia, and depression,^23^ because these complications would be investigated as outcomes or were correlated with the outcome variables in this study. These complications were identified by examining the clinical diagnosis history of each COPD patient. Patients without any records of public health care service attendance during the entire follow-up period were considered as lost to follow-up and thus were excluded from analysis. Furthermore, patients who attended highly related programs operated by nurses and/or allied health workers, including the Integrated Mental Wellness Program and NAHC-Medication Management, were also excluded to avoid contamination with the outcomes from other NAHC programs.

### Exposure Variable

The exposure variable was whether the patient first joined NAHC-Respiratory during 2010 to 2014. The exposure group (NAHC group) included patients who joined NAHC-Respiratory while also receiving usual care during 2010 to 2014, and the reference group (usual care only group) included patients who did not join NAHC-Respiratory while receiving usual care during 2010 to 2019. Patients who did not join NAHC-Respiratory during 2010 to 2014 but joined during 2015 to 2019 were excluded due to insufficient follow-up duration. The index date was defined as the first attendance date in the NAHC-Respiratory program (for the exposure group) or general outpatient, family medicine, specialist outpatient services for COPD follow-up (for the reference group) to avoid immortal time bias, as the index date was defined as the date of recording the exposure factor (ie, attending NAHC-Respiratory). The end of study follow-up was determined as December 31, 2019, based on data availability in the HADCL when initiating the study.

### Outcome Variables

Outcome events involved all-cause mortality, cause-specific mortality, incidence of common complications, and health care service use. All-cause and cause-specific mortality were the primary outcomes, including death caused by COPD, pneumonia, respiratory failure, lung cancer, all respiratory conditions, and all cardiovascular conditions, with the underlying cause of death identified based on *ICD-10-CM* codes (eTable 1 in Supplement 1). Incidence of pneumonia, respiratory failure, lung cancer, pulmonary heart disease, pneumothorax, anemia, polycythemia, and depression were included as secondary outcomes.^23^ For health care service use, we calculated rates of emergency department attendance, hospitalization, hospitalization through the emergency department (emergent hospitalization), and intensive care unit (ICU) or high-dependency unit (HDU) admission.

### Baseline Covariates

A literature review was conducted to synthesize covariates used in studies for patients with COPD, which were considered for further statistical adjustment in our study. The baseline covariates included age; sex; whether or not receiving social security assistance; whether or not living in an elderly home; baseline smoking status; diagnosis of asthma, bronchiectasis, tuberculosis, or hypertension; Charlson Comorbidity Index (CCI, but excluding COPD itself); duration since diagnosis of COPD; body mass index (calculated as weight in kilograms divided by height in meters squared); vaccination against pneumococcus; previous use of noninvasive ventilation; long-term oxygen use; previous use of bronchodilators, systemic glucocorticoids, short- or long-acting β2-adrenergic agonists, or short- or long-acting muscarinic antagonists; previous attendance at any other NAHC service (wound care, fall prevention, or continence care), day rehabilitation services, Risk Assessment and Management Program (RAMP) for diabetes or hypertension, or Smoking Counseling and Cessation Progamme (SCCP); and whether or not hospitalized 3 years prior to index date.^13,24,25,26^ The comorbidities, duration since COPD diagnosis, use of relevant clinical procedures, and use of medications were used to indicate the severity of COPD. The use of antibiotics was not included as their use is not specific to respiratory infections only; some antibiotics for respiratory tract infections can also be used for other conditions, such as urinary tract infections. Blood eosinophil or neutrophil count was not included since it was not available for approximately half of the patients at baseline. Attendance at other NAHC services, RAMP, and SCCP partially served as proxies of patients’ willingness to attend primary care chronic disease management services and their motivations on self-care for their own chronic conditions and thus were included as a covariate to be matched.

### Propensity Score Matching

To obtain comparable subsets of patients between the NAHC group and usual care group, a propensity score matching procedure was performed. Individuals with missing values in baseline variables were excluded. A multivariate logistic regression was performed for all baseline factors of the NAHC vs usual care groups, and the regression coefficients were used to estimate the probabilities for each individual to be an NAHC recipient (ie, propensity score). A nearest-neighbor-joining matching with a caliper of 0.05-fold of standard deviation was adopted to select participants in the NAHC group and usual care group on a 1:2 ratio with discard for both groups. The absolute standardized mean difference (ASMD) was estimated for each baseline variable. A threshold of 0.1 was adopted for ASMD to determine whether a baseline variable is satisfactorily balanced after matching.

### Statistical Analysis

Baseline covariates were described before and after propensity score matching. Statistical analysis was then conducted using the after-matching dataset. All-cause mortality rate, cause-specific mortality rate, disease incidence, and mean number of health care service attendance were estimated for the NAHC group and usual care group separately. Crude absolute risk reduction and incidence rate ratio (IRR) were calculated. Kaplan-Meier curves were plotted for time to all-cause mortality for the 2 groups. For all-cause mortality, Cox proportional hazard regression was applied to estimated hazard ratios (HRs) between the 2 groups. The proportional hazard assumption was tested by using the Schoenfeld global test. For cause-specific mortality and disease incidence, a competing risk regression with subdistribution hazard function described in Fine and Gray^27^ was used to estimate the HR to account for multiple outcomes by using the R package “cmprsk.”^28^ For health care service use, overdispersion Poisson log-link regression was used to estimate the risk ratio of number of times of using the health care services, and log-link gamma regression model was used to estimate the risk ratio of length of stay. The offset term for these regression models was the natural logarithm of the length of time at risk for each outcome event. For the outcome variable as the number of emergency department visits and hospitalizations, the length of time at risk was the length of time staying outside the inpatient wards (ie, total follow-up time − total length of stay at inpatient wards). For the outcome variable as the number of ICU/HDU admissions, the length of time at risk was the length of time staying outside the ICD/HDU (ie, total follow-up time − total length of stay at ICU/HDU). For modeling the length-of-stay outcomes, the length of time at risk was total follow-up time.

The statistical uncertainty was assessed by using 95% CIs and *P* value of model estimates. The 95% CI was constructed by using the point estimate and standard error calculated using the delta method. The *P* value was calculated using the 2-sided Wald test. Statistical significance was claimed when the *P* value was less than .05.

Two sensitivity analyses were conducted. First, a sensitivity analysis was conducted to include patients with outcome events occurring more than 2 years after the index date to avoid reverse causality between attendance of NAHC and health outcomes. Second, an area variable that uses median household income to stratify the districts in Hong Kong evenly into 3 levels^29^ was added to the regression models as additional covariates in 2 dummy variables to adjust for potential confounders associated with areas and socioeconomic status. Subgroup analysis was performed for patients with different age groups (<60, 60-79, and ≥80 years), smoking status (whether or not an ever smoker), and CCI score (CCI = 0 or >0) at baseline. Moreover, as spirometry measurements were not included as covariates due to a high proportion of missing values, a tipping point analysis on the robustness of the results to an unobserved spirometry confounder (ie, FEV_1_) was conducted using the R package tipr (R Project for Statistical Computing).^30,31^ All statistical analysis was conducted between August 2023 and April 2024, in R statistical software, version 3.6.1 (R Project for Statistical Computing).

## Results

A total of 38 351 patients with COPD and who attended outpatient clinics for COPD between 2010 and 2014 were identified (eFigure 1 in Supplement 1). Among them, 3121 eligible patients without missing values in baseline variables participated in NAHC-Respiratory during 2010 to 2014 (NAHC group), and there were 17 316 eligible patients without missing values in baseline variables who did not attend NAHC-Respiratory throughout 2010 to 2019 (usual care group). After propensity score matching, 3093 patients in the NAHC group and 5955 patients in the usual care group were selected, for an overall sample size of 9048. Comparisons of baseline characteristics between individuals with and without any missing values and between matched and unmatched individuals are shown in eTable 2 in Supplement 1.

Table 1 shows the baseline characteristics of patients in the 2 groups. At baseline, ASMDs were lower than 0.1 for all baseline covariates after matching. There were 2814 (91.0%) and 5431 (91.2%) men, 279 (9.0%) and 524 (8.8%) women, 822 (26.6%) and 1596 (26.8%) public assistance recipients, and 1418 (45.9%) and 2673 (44.9%) known smokers in the NAHC group and usual care group, respectively. Mean (SD) age at baseline was 69.8 (9.5) years in the NAHC group and 69.5 (11.7) years in the usual care group. Mean (SD) duration between COPD diagnosis and index date was 0.6 (1.9) years for both groups.

**Table 1.** Sample Characteristics

Baseline variable	Patients, No. (%)					
	Before matching			After matching		
	NAHC (n = 3121)	Usual care (n = 17 316)	ASMD	NAHC (n = 3093)	Usual care (n = 5955)	ASMD
**Sociodemographics and lifestyle**						
Sex						
Male	2842 (91.1)	13982 (80.8)	0.262	2814 (91.0)	5431 (91.2)	0.008
Female	279 (8.9)	3334 (19.3)	0.262	279 (9.0)	524 (8.8)	0.008
Age at baseline, mean (SD), y	69.7 (9.5)	72.0 (11.2)	0.198	69.8 (9.5)	69.5 (11.7)	0.025
Calendar year of cohort enrollment	2012	2011	0.404	2012	2012	0.015
Elderly home residence	37 (1.2)	597 (3.5)	0.124	37 (1.2)	64 (1.1)	0.011
Receipt of public assistance	829 (26.6)	4728 (27.3)	0.017	822 (26.6)	1596 (26.8)	0.005
Smoking status						
Ever smoker	1446 (46.3)	5701 (32.9)	0.285	1418 (45.9)	2673 (44.9)	0.019
Unknown	1620 (51.9)	10788 (62.3)	0.214	1620 (52.4)	3156 (53.0)	0.012
**Clinical characteristics**						
BMI, mean (SD)	23.1 (3.9)	23.3 (4.1)	0.059	23.1 (3.9)	23.1 (4.0)	0.003
Asthma	324 (10.4)	2010 (11.6)	0.038	323 (10.4)	618 (10.4)	0.002
Bronchiectasis	25 (0.8)	404 (2.3)	0.101	25 (0.8)	46 (0.8)	0.004
Tuberculosis	62 (2.0)	469 (2.7)	0.044	62 (2.0)	116 (2.0)	0.004
Hypertension	1596 (51.1)	8776 (50.7)	0.009	1576 (51.0)	3052 (51.2)	0.006
Charlson Comorbidity Index, mean (SD)	0.4 (0.9)	0.5 (1.1)	0.131	0.4 (0.9)	0.4 (0.8)	0.005
Vaccination against pneumococcal disease	832 (26.7)	3672 (21.2)	0.133	819 (26.5)	1518 (25.5)	0.023
Duration since COPD diagnosis, mean (SD), y	0.6 (1.9)	0.7 (2.0)	0.023	0.6 (1.9)	0.6 (1.9)	0.001
Use of long-term oxygen	0	26 (0.2)	0.039	0	0	0.000
Use of noninvasive ventilation	5 (0.2)	77 (0.4)	0.043	5 (0.2)	13 (0.2)	0.014
**Use of medications**						
Long-acting β2 agonists	77 (2.5)	639 (3.7)	0.065	77 (2.5)	133 (2.2)	0.018
Long-acting muscarinic antagonist	30 (1.0)	219 (1.3)	0.027	30 (1.0)	52 (0.9)	0.010
Phosphodiesterase inhibitors	855 (27.4)	5609 (32.4)	0.107	853 (27.6)	1575 (26.5)	0.026
Short-acting β2 agonists	2555 (81.9)	15 378 (88.8)	0.220	2544 (82.3)	4886 (82.1)	0.005
Short-acting muscarinic antagonist	774 (24.8)	5070 (29.3)	0.098	767 (24.8)	1462 (24.6)	0.006
Systemic glucocorticoids	1640 (52.6)	10 430 (60.2)	0.157	1634 (52.8)	3081 (51.8)	0.022
**Health care service utilization**						
Ever hospitalized in past 3 y	1198 (38.4)	8978 (51.9)	0.269	1194 (38.6)	2269 (38.1)	0.010
Attendance of any RAMP services for diabetes and hypertension	77 (2.5)	179 (1.0)	0.142	73 (2.4)	141 (2.4)	0.000
Attendance of day rehabilitation services	38 (1.2)	303 (1.8)	0.041	36 (1.2)	91 (1.5)	0.030
Attendance of other NAHC services	21 (0.7)	75 (0.4)	0.037	21 (0.7)	39 (0.7)	0.002
Attendance of smoking cessation services	179 (5.7)	281 (1.6)	0.325	162 (5.2)	281 (4.7)	0.024

Abbreviations: ASMD, absolute standardized mean difference; BMI, body mass index (calculated as weight in kilograms divided by height in meters squared); COPD, chronic obstructive pulmonary disease; NAHC, Nurse and Allied Health Clinic; RAMP, Risk Assessment and Management Programme.

During the follow-up period, 32.1% (n = 994) and 37.2% (n = 2212) of patients died in the NAHC group (median [IQR] follow-up, 7.3 [5.5-8.6] years) and usual care group (median follow-up, 6.8 [5.3-8.7] years), respectively (Table 2). The all-cause mortality rate was 47.2 and 56.2 per 1000 patient-years for the NAHC and usual care groups, respectively. The Kaplan-Meier curve showed that patients in the NAHC group had a lower all-cause mortality risk than the usual care group (Figure). Cox regression estimation showed that attending NAHC-Respiratory was associated with lower all-cause mortality risk (HR, 0.84; 95% CI, 0.78-0.90). Among patients with different causes of death, attending NAHC-Respiratory was associated with a lower mortality risk caused by pneumonia (HR, 0.85; 95% CI, 0.74-0.97), any respiratory condition (HR, 0.86; 95% CI, 0.77-0.96), and any cardiovascular condition (HR, 0.74; 95% CI, 0.59-0.93).

**Table 2.** Mortality Outcomes for 3093 NAHC vs 5955 Usual Care Patients^a^

Outcome	Cumulative mortality, No. (%)	Rate per 1000 patient-years	ARR, %	HR (95% CI)	*P* value	
**All-cause mortality**						
Usual care group	2212 (37.2)	56.15	NA	1 [Reference]	NA	
NAHC group	994 (32.1)	47.15	9.00	0.84 (0.78-0.90)^b^	<.001	
**Cause-specific mortality**						
COPD						
Usual care group	240 (4.0)	6.09	NA	1 [Reference]	NA	
NAHC group	112 (3.6)	5.31	0.78	0.90 (0.72-1.12)	.35	
Pneumonia						
Usual care group	673 (11.3)	17.08	NA	1 [Reference]	NA	
NAHC group	298 (9.6)	14.14	2.95	0.85 (0.74-0.97)^b^	.02	
Respiratory failure						
Usual care group	36 (0.6)	0.91	NA	1 [Reference]	NA	
NAHC group	21 (0.7)	1.00	−0.08	1.12 (0.65-1.92)	.68	
Lung cancer						
Usual care group	250 (4.2)	6.35	NA	1 [Reference]	NA	
NAHC group	129 (4.2)	6.12	0.23	0.99 (0.80-1.22)	.91	
Any respiratory condition						
Usual care group	1000 (16.8)	25.38	NA	1 [Reference]	NA	
NAHC group	450 (14.6)	21.35	4.04	0.86 (0.77-0.96)^b^	.009	
Any cardiovascular condition						
Usual care group	258 (4.3)	6.55	NA	1 [Reference]	NA	
NAHC group	100 (3.2)	4.74	1.81	0.74 (0.59-0.93)^b^	.009	
Any cancer						
Usual care group	506 (8.5)	12.84	NA	1 [Reference]	NA	
NAHC group	250 (8.1)	11.86	0.99	0.94 (0.81-1.10)	.43	

Abbreviations: ARR, absolute risk reduction; COPD, chronic obstructive pulmonary disease; HR, hazard ratio; NA, not applicable; NAHC, Nurse and Allied Health Clinic.

^a^ The median follow-up period was 7.3 years for the NAHC group and 6.8 years for the usual care group.

^b^ *P* < .05.

**Figure.**
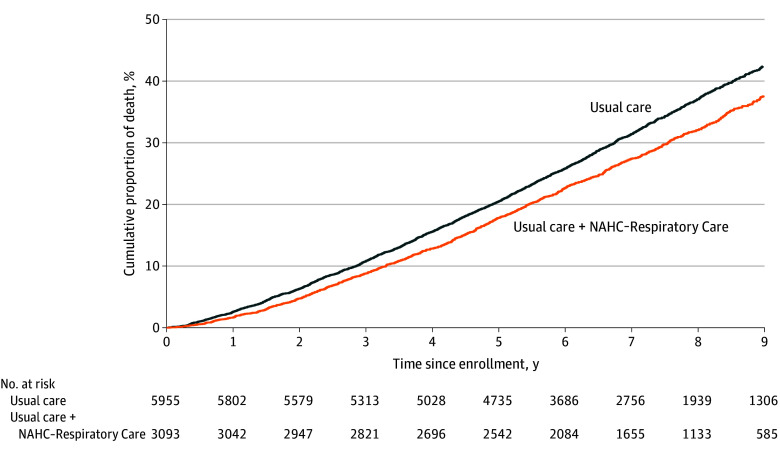
Kaplan-Meier Curve for All-Cause Mortality in the Nurse and Allied Health Clinic (NAHC) Group (n = 3093) vs Usual Care Group (n = 5955)

Sensitivity analysis on patients with outcome events that occurred at least 2 years after the index date showed similar patterns of the mortality risks, where using NAHC-Respiratory was associated with a relatively lower level of all-cause mortality risk (HR, 0.86; 95% CI, 0.79-0.93) and mortality risk caused by pneumonia (HR, 0.84; 95% CI, 0.72-0.97), any respiratory condition (HR, 0.85; 95% CI, 0.75-0.96), and any cardiovascular condition (HR, 0.76; 95% CI, 0.59-0.98) among patients alive for at least 2 years after the index date (eTable 3 in Supplement 1). The sensitivity analysis additionally adjusted for the area-level covariate in the regression model and found that the associations between NAHC-Respiratory attendance and different outcomes were similar between the models with and without the adjustment of the area variable (eTable 4 in Supplement 1). The association between attending NAHC-Respiratory and all-cause mortality risk differed across subgroups (Table 3). The association was statistically significant in patients aged 60 to 79 years at baseline (HR, 0.82; 95% CI, 0.74-0.90) and ever smokers (HR, 0.75; 95% CI, 0.68-0.84) and was stronger in patients with a CCI of 1 or greater (HR, 0.74; 95% CI, 0.80-0.96) than those with a CCI of 0 (HR, 0.88; 95% CI, 0.80-0.96) (CCI excluding COPD itself). The association was not significant for patients younger than 60 years, 80 years or older, or who were not smokers or without smoking status records. The Kaplan-Meier curves for all-cause mortality stratified by age groups, smoking status, and CCI scores can be found in eFigures 2-4 in Supplement 1, respectively.

**Table 3.** Subgroup Results for All-Cause Mortality

Subgroup	All-cause mortality			ARR, %	Cox regression estimation	
	Patients, No.	Cumulative mortality, No. (%)	Rate per 1000 patient-years		HR (95% CI)	*P* value
Age at baseline						
<60 y						
Usual care group	1004	101 (10.1)	13.96	NA	1 [Reference]	NA
NAHC group	455	47 (10.3)	13.70	0.27	0.97 (0.68-1.37)	.86
60-79 y						
Usual care group	3742	1388 (36.0)	53.14	NA	1 [Reference]	NA
NAHC group	2068	645 (30.1)	43.46	9.68	0.82 (0.74-0.90)	<.001
≥80 y						
Usual care group	1090	723 (66.3)	119.64	NA	1 [Reference]	NA
NAHC group	497	302 (60.8)	107.47	12.17	0.91 (0.79-1.04)	.15
Smoking status, ever smoker						
No or unknown						
Usual care group	3368	1130 (33.6)	48.83	NA	1 [Reference]	NA
NAHC group	1675	520 (31.0)	44.36	4.47	0.91 (0.82-1.01)	.08
Yes						
Usual care group	2587	1082 (41.8)	66.57	NA	1 [Reference]	NA
NAHC group	1418	1082 (33.4)	50.64	15.93	0.75 (0.68-0.84)	<.001
CCI score						
0						
Usual care group	4630	1545 (33.4)	49.25	NA	1 [Reference]	NA
NAHC group	2425	726 (29.9)	43.25	6.00	0.88 (0.80-0.96)	.004
≥1						
Usual care group	1325	667 (50.3)	83.15	NA	1 [Reference]	NA
NAHC group	668	268 (40.1)	62.38	20.77	0.74 (0.64-0.85)	<.001

Abbreviations: ARR, absolute risk reduction; CCI, Charlson Comorbidity Index; HR, hazard ratio; NA, not applicable; NAHC, Nurse and Allied Health Clinic.

Robustness of the regression results on all-cause mortality to unobserved FEV_1_ was tested. While 93.6% of the patients in the matched cohort did not have a baseline FEV_1_ value recorded, the percentage with severe impaired FEV_1_ among remaining patients was 24.4% in the exposure group and 22.7% in the reference group. The HR of all-cause mortality for patients with severe impaired FEV_1_ group vs no impairment group was assumed to be 2.54 based on a recent cohort study.^3^ In the tipping point analysis, the HR for all-cause mortality of the NAHC group vs the usual care group adjusted by inclusion of the observed FEV_1_ was estimated as 0.81 (95% CI, 0.76-0.89), which was similar to the primary outcome of 0.84 (95% CI, 0.78-0.90). Moreover, it was found that the percentage of severe impaired FEV_1_ in the reference group needed to reach 34.0% to make the upper limit of the HR (ie, 0.90 reach 1), keeping the percentage in the exposure group unchanged (24.4%). In another scenario, the percentage of severe impaired FEV_1_ in the exposure group needs to reach 15% to make the upper limit of the HR (ie, 0.90) reach 1, keeping the percentage in the exposure group unchanged (22.7%).

For secondary outcomes, attending NAHC-Respiratory was not significantly associated with incidence risk of any of the selected COPD complications (eTable 5 in Supplement 1). Attending NAHC-Respiratory was associated with reduced use of hospital care services. It was associated with reduced rates of emergency department attendance (IRR, 0.92; 95% CI, 0.86-0.98) and emergent hospitalization (IRR, 0.89; 95% CI, 0.83-0.95) as well as reduced length of stay of overall inpatient service (IRR, 0.90; 95% CI, 0.84-0.98) and emergent hospitalization (IRR, 0.82; 95% CI, 0.75-0.88) (Table 4).

**Table 4.** Health Care Use Outcomes for NAHC vs Usual Care

Outcome	Mean (SD)	Rate per patient-year	Healthcare service use	
			IRR (95% CI)	*P* value
**No. of admissions**				
Emergency department				
Usual care group	7.17 (10.60)	1.10	1 [Reference]	NA
NAHC group	6.81 (9.06)	1.02	0.92 (0.86-0.98)	.02
Hospitalization				
Usual care group	7.35 (11.42)	1.13	1 [Reference]	NA
NAHC group	7.17 (8.69)	1.07	0.95 (0.88-1.01)	.10
Emergent hospitalization				
Usual care group	4.39 (6.41)	0.68	1 [Reference]	NA
NAHC group	4.03 (5.62)	0.60	0.89 (0.83-0.95)	.001
ICU or HDU admission				
Usual care group	0.04 (0.22)	0.006	1 [Reference]	NA
NAHC group	0.03 (0.20)	0.005	0.84 (0.52-1.35)	.4
**Total length of stay, d**				
Hospitalization				
Usual care group	44.71 (66.75)	6.76	1 [Reference]- -	NA
NAHC group	41.62 (66.96)	6.11	0.90 (0.84-0.98)	.01
Emergent hospitalization				
Usual care group	26.62 (42.38)	4.02	1 [Reference]	NA
NAHC group	22.38 (33.75)	3.28	0.82 (0.75-0.88)	<.001
ICU or HDU admission				
Usual care group	0.29 (2.64)	0.044	1 [Reference]	NA
NAHC group	0.24 (2.27)	0.036	0.82 (0.37-1.78)	.61

Abbreviations: HDU, high-dependency unit; ICU, intensive care unit; IRR, incidence rate ratio; NA, not applicable; NAHC, Nurse and Allied Health Clinic.

## Discussion

This is, to our knowledge, one of the first studies reporting the mortality, morbidity, and service use outcomes of patients with COPD using a multidisciplinary COPD treatment and rehabilitation program coordinated by nurses, physiotherapists, and occupational therapists. The findings showed that the patients attending the NAHC-Respiratory program had a more than 10% lower risk for all-cause mortality as well as mortality caused by pneumonia, any respiratory conditions, or any cardiovascular conditions. It was also highlighted in the outcomes that attending this program was associated with reduced emergency department use and hospitalization, whereas there was no significant difference found for incidence risk of a few common complications of COPD.

The NAHC-Respiratory program involves patient education on physical exercise and lifestyle, smoking cessation, vaccinations, and pulmonary rehabilitation for patients with poor lung function. Articles on other integrated COPD management or self-management interventions identified in the literature did not report the mortality outcomes in their cohorts.^14,16,17,18^ Our study reported similar outcomes in terms of reduction of mortality and hospitalization risks among patients with COPD as those in previous studies on pulmonary rehabilitation, while the magnitude of relative risk reduction of mortality reported in these studies (range, 31%-42%) was larger than for the NAHC-Respiratory program.^32,33,34,35^ This difference may primarily be caused by differences in their service packages and how patients were targeted. The NAHC-Respiratory program provided a mixture of self-management education, risk factor midifications, and rehabilitation services to patients with COPD at primary care settings whose baseline mortality risk was relatively low, while the pulmonary rehabilitation services reported in the literature primarily targeted more high-risk patients following their excerbations and hospitalizations with a baseline annual mortality rate ranging from 17.3% to 25.2%.^32,34,35^

Nevertheless, the lower mortality risk of NAHC-Respiratory participants in more than 6 years of follow-up implies its long-term benefits in health outcomes, particularly for death caused by pneumonia, respiratory conditions, and cardiovascular conditions. This benefit may be attributed to seasonal influenza and pneumococcal vaccinations and smoking cessation, which are 2 of the services provided in the program for all attendees. It was reported elsewhere that 42% and 40% of patients with COPD who were treated in the NAHC program received a seasonal influenza vaccine during 2016 to 2017 and a pneumococcal vaccine before 2017, respectively, and these rates improved to 49% and 57% for another cohort of patients in 2018 to 2019.^22^ The pneumococcal vaccination rate was higher than those of people older than 65 years in Hong Kong, which was 33.9% in 2015 to 2016 and 38.2% in 2017 to 2018.^36^ According to previous studies, while pneumococcal vaccination may not be able to significantly reduce the incidence of pneumonia among older individuals,^37^ it can considerably reduce risk of death and hospitalization caused by all-cause pneumonia or lower respiratory infections.^37,38^ This could lead to reduction of severe cases and death caused by pneumonia among patients with COPD.

Smoking cessation could be another reason for the improvement of health outcomes. The greater reduction in all-cause mortality among ever smokers in subgroup analysis supports this explanation (eFigure 3 in Supplement 1). The attendance at smoking cessation service was 44% in 2016 to 2017 and 48% in 2018 to 2019, respectively.^22^ Although information on patients successfully quitting smoking was not recorded in the dataset, it was reported by the Hospital Authority that 46% (in 2010) and 51% (in 2013) of patients who had attended the smoking cessation services had quit smoking at 52 weeks after enrollment.^39^ The high quitting rates suggest that referral to smoking cessation services by NAHC-Respiratory may be associated with lower mortality caused by respiratory and cardiovascular conditions in patients with COPD.^40,41,42^

The health benefits associated with use of NAHC-Respiratory, including reduction in use of hospital care services, may also be attributed to health education (including assessment by nurses on inhaler compliance and technique) and pulmonary rehabilitation for the patients. Systematic reviews have summarized that health education on self-management of COPD is associated with better health status in terms of a lower St George’s Respiratory Questionnaire score, improved mental status, and lower use of inpatient services,^14,43,44,45^ and self-management education was found to be more influential for patients’ well-being than provision of exercise information and formulation of an action plan for exacerbation.^14^ These health education activities were helpful in improving breathing techniques, enhancing knowledge of COPD, increasing self-efficacy in disease management, and alleviating patients’ emotional problems, although they were not associated with improved lung function.^14,44,45^ Apart from self-management education, a brief pulmonary rehabilitation program for patients with COPD with severe respiratory symptoms and poorer lung function may be another reason for the observed health improvements. A meta-analysis has shown that long-term, supervised pulmonary rehabilitation programs are associated with improved quality of life and lung function performance of patients with COPD.^15^ In addition, a 7-week pulmonary rehabilitation intervention involving aerobic training, behavioral therapy, health education, and smoking cessation was found to be associated with a lower level of cardiovascular risk factors, including aortic pulse wave velocity, blood pressure, total cholesterol, and interleukin-6 level.^46^ This could partially explain why attending the NAHC-Respiratory program was associated with cardiovascular benefits.

Compared with other integrated COPD management programs in the primary care setting reported in the literature, there are several shared components among all these programs, including NAHC-Respiratory, such as education on COPD self-management skills and lifestyle, exercise training, energy conservation and breathing techniques, smoking cessation, and formulating individualized action plans for COPD management.^16,17,18^ However, on one hand, not every other program directly provided pneumococcal or seasonal influenza vaccinations like the NAHC-Respiratory did, which may be one of the key reasons why patients in NAHC-Respiratory had lower risk for pneumonia mortality. On the other hand, unlike other programs, the current NAHC-Respiratory protocol does not universally involve telemonitoring of patients’ physical activities,^16^ which could potentially improve the quality of care. Moreover, it was also shown in a systematic review that pulmonary rehabilitation was more beneficial in home-based settings and for patients with a less severe condition (FEV_1_:FVC >50%),^15^ which suggests that NAHC-Respiratory might also consider incorporating home-based rehabilitation elements as well as shifting the target to patients with milder lung function impairment.

### Limitations

There were limitations to this study. First, although ambulatory patients with COPD were referred by health care professionals to the NAHC-Respiratory program in Hong Kong for primary care treatment, patients in the NAHC-Respiratory program might be more motivated to care for their own health conditions since they voluntarily attended this program for chronic disease management in addition to usual care. Nevertheless, baseline characteristics used for propensity score matching involved the use of other primary care or rehabilitation services, which could be proxies for their health literacy and practice of accessing health care service, and were balanced in the matched groups. Furthermore, as a common limitation for all observational studies, the disparities in participants’ baseline characteristics before matching cannot be fully addressed by merely balancing the observable covariables during matching, which still left some of the unobservable covariables unbalanced. As such, a randomized clinical trial may be required to examine the causal effect of the NAHC-Respiratory program on adverse health outcomes among ambulatory patients with COPD. Second, spirometry data and other lung function measurements were not sufficiently recorded in the database, particularly for patients in the reference group. Therefore, they were not included as outcomes or baseline characteristics in the analysis. We have conducted a tipping point analysis on the robustness of the results to this unobserved confounder using the R package tipr.^30,31^ The tipping point analysis found that the adjusted HR for all-cause mortality by inclusion of FEV_1_ was similar to the primary outcome. It also found that the upper limit of the HR would not reach 1 unless there was a substantial gap in percentage of patients with severe impaired FEV_1_ between the exposure group and the reference group. Considering that we used other variables to match the severity of COPD and health status at baseline, including comorbidities, duration since COPD diagnosis, clinical procedures, and use of medications, having such a large difference in the percentage of severe impaired FEV_1_ is unlikely. Therefore, the missing FEV_1_ values at baseline are unlikely to affect the statistical significance of the association between NAHC attendance and all-cause mortality. Finally, the database used electronic health records in the public health care system. While most patients with COPD were treated in public hospitals and clinics, a few services that are predominately offered in the private sector were not recorded, such as vaccination against influenza among people younger than 65 years.

## Conclusions

To our knowledge, this population-based cohort study is the first to examine the medium- or long-term health outcomes among patients who joined a protocol-driven, nurse- and allied health–led, multidisciplinary COPD management program. The study outcomes highlight reduced mortality risk and use of hospital care services among the program users, particularly for patients aged 60 to 79 years and ever smokers. These findings indicate the program’s potential to improve long-term health outcomes of patients with COPD and save health care resources of hospitals through a primary care intervention, although improvement of service provision may be needed for patients younger than 60 years. This study provides evidence that the care model involving nurses and allied health professionals can facilitate COPD treatment, highlighting that health care professionals other than physicians are important in patient follow-up and disease management in the primary care setting. The program components can be considered in addition to usual care or can be used to inform the design of other related programs to benefit more patients. In the future, experimental studies can be conducted to examine the program’s effectiveness on health outcomes, and implementation science studies can be considered to improve its service delivery.

## Notes

Supplement 1. **eFigure 1.** Flow chart for inclusion and exclusion of patients **eFigure 2.** Kaplan-Meier curve for all-cause mortality in NAHC group versus usual care only group stratified by age group at baseline **eFigure 3.** Kaplan-Meier curve for all-cause mortality in NAHC group versus usual care only group stratified by smoking status **eFigure 4.** Kaplan-Meier curve for all-cause mortality in NAHC group versus usual care only group stratified by Charlson Comorbidity Index (CCI) score **eTable 1.** The ICD-10-CM, and ICPC-2 codes used to define the clinical diagnoses **eTable 2.** Comparison of baseline characteristics between individuals with and without any missing value at baseline, and between matched and unmatched individuals **eTable 3.** Sensitivity analysis on risk of mortality among patient alive for at least 2 years after index date **eTable 4.** The comparison of the regression results between models with and without adjustment of an additional area variable **eTable 5.** Risk of diseases incidence in NAHC group versus usual care only group

Supplement 2.
Data Sharing Statement
