# Supplementary material for: Outcomes and Hospital Service Use Among Patients With COPD in a Nurse- and Allied Health–Led Clinic
Source: JAMA Health Forum. 2024 Jul 5;5(7):e241575. doi: 10.1001/jamahealthforum.2024.1575 (PMC11227079; doi:10.1001/jamahealthforum.2024.1575)
Supplement: Supplement 1. — eFigure 1. Flow chart for inclusion and exclusion of patients eFigure 2. Kaplan-Meier curve for all-cause mortality in NAHC group versus usual care only group stratified by age group at baseline eFigure 3. Kaplan-Meier curve for all-cause mortality in NAHC group versus usual care only group stratified by smoking status eFigure 4. Kaplan-Meier curve for all-cause mortality in NAHC group versus usual care only group stratified by Charlson Comorbidity Index (CCI) score eTable 1. The ICD-10-CM, and ICPC-2 codes used to define the clinical diagnoses eTable 2. Comparison of baseline characteristics between individuals with and without any missing value at baseline, and between matched and unmatched individuals eTable 3. Sensitivity analysis on risk of mortality among patient alive for at least 2 years after index date eTable 4. The comparison of the regression results between models with and without adjustment of an additional area variable eTable 5. Risk of diseases incidence in NAHC group versus usual care only group [file jamahealthforum-e241575-s001.pdf]

Supplemental Online Content

Wang K, Zhao S, Yau SZM, et al. Outcomes and hospital service use among patients with COPD in a nurse- and allied health–led clinic. *JAMA Health Forum*. 2024;5(6):e241575. doi:10.1001/jamahealthforum.2024.1575

eFigure 1. Flow chart for inclusion and exclusion of patients

eFigure 2. Kaplan-Meier curve for all-cause mortality in NAHC group versus usual care only group stratified by age group at baseline

eFigure 3. Kaplan-Meier curve for all-cause mortality in NAHC group versus usual care only group stratified by smoking status

eFigure 4. Kaplan-Meier curve for all-cause mortality in NAHC group versus usual care only group stratified by Charlson Comorbidity Index (CCI) score

eTable 1. The ICD-10-CM, and ICPC-2 codes used to define the clinical diagnoses

eTable 2. Comparison of baseline characteristics between individuals with and without any missing value at baseline, and between matched and unmatched individuals

eTable 3. Sensitivity analysis on risk of mortality among patient alive for at least 2 years after index date

eTable 4. The comparison of the regression results between models with and without adjustment of an additional area variable

eTable 5. Risk of diseases incidence in NAHC group versus usual care only group

This supplemental material has been provided by the authors to give readers additional information about their work.

© 2024 Wang K et al. *JAMA Health Forum.*

eFigure 1. Flow chart for inclusion and exclusion of patients

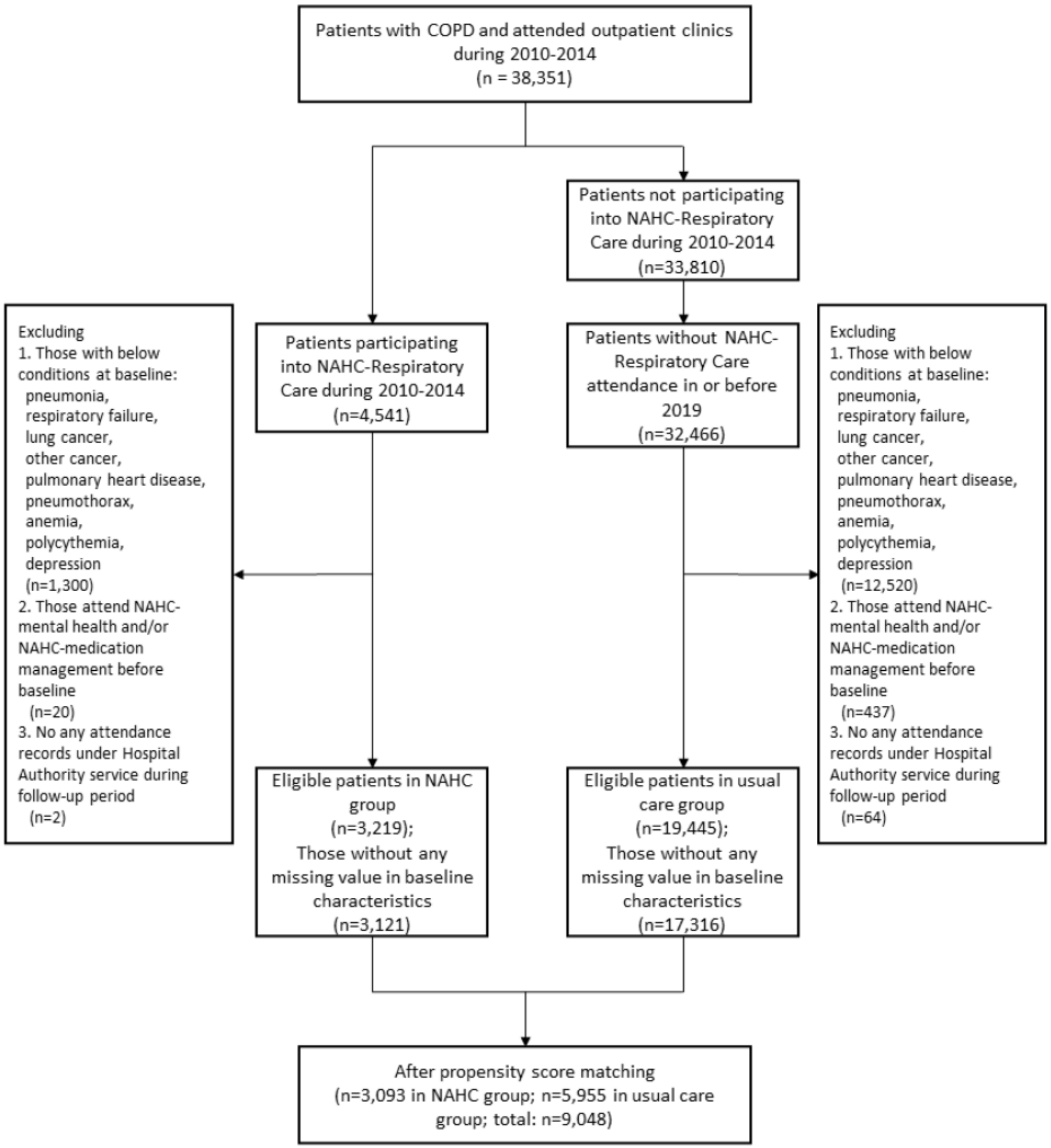

© 2024 Wang K et al. *JAMA Health Forum.*

eFigure 2. Kaplan-Meier curve for all-cause mortality in NAHC group versus usual care only group stratified by age group at baseline

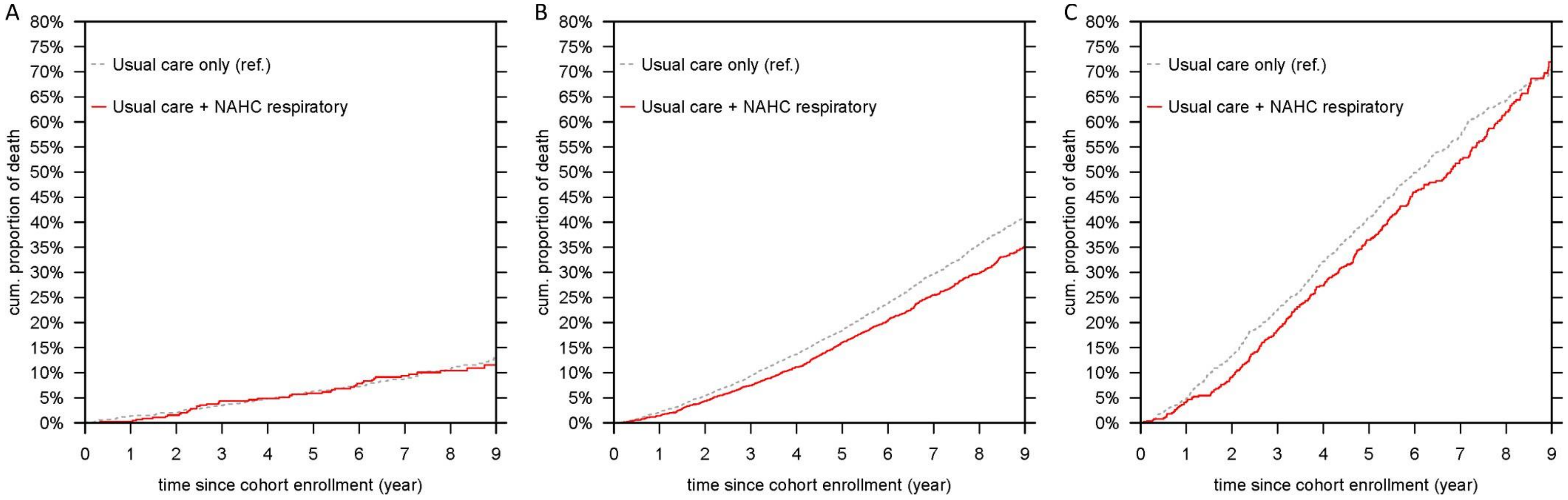

Note: Panel A: Age at baseline < 60 years; Panel B: Age at baseline ≥ 60 years & < 80 years; Panel C: Age at baseline ≥ 80 years.

© 2024 Wang K et al. *JAMA Health Forum.*

eFigure 3. Kaplan-Meier curve for all-cause mortality in NAHC group versus usual care only group stratified by smoking status

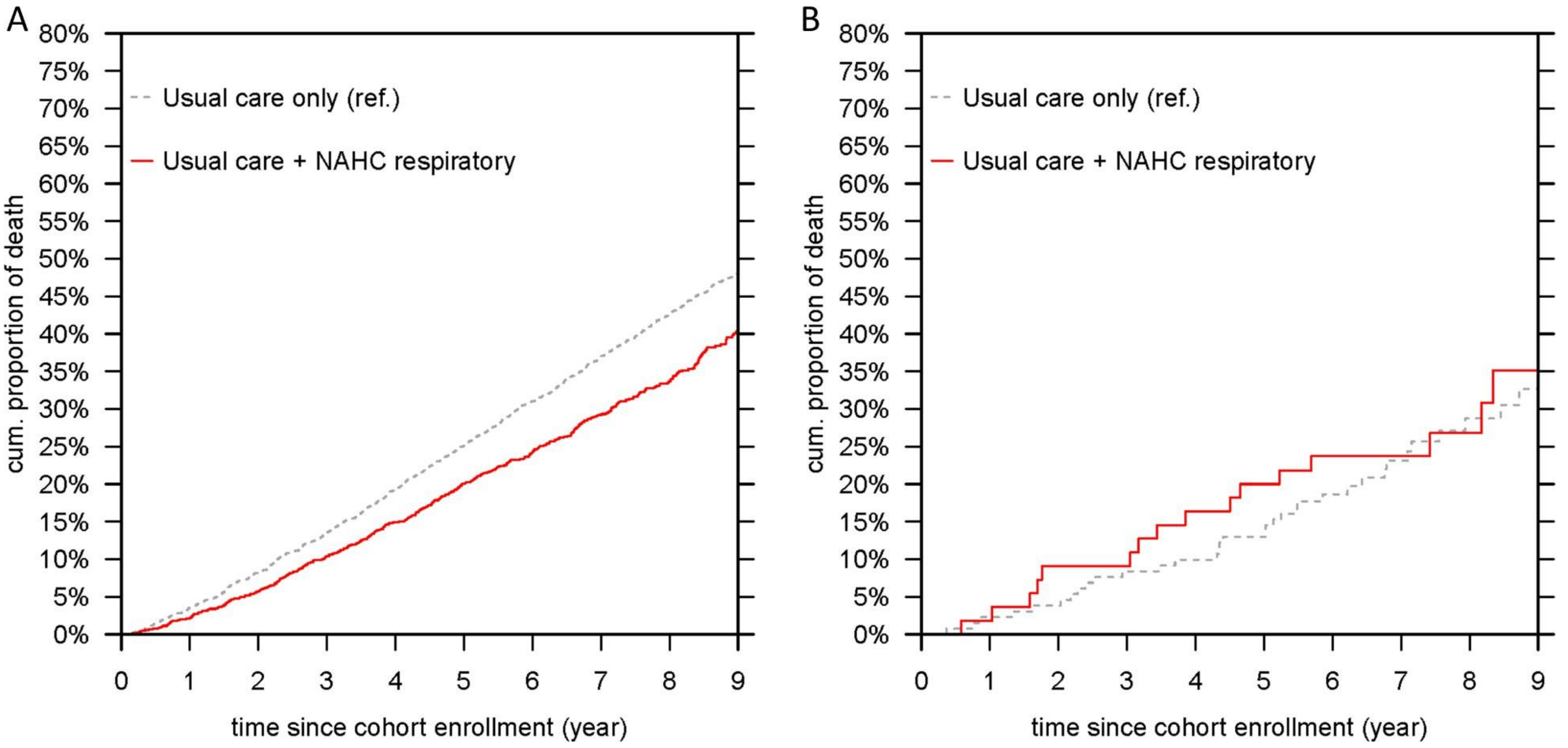

Note: Panel A: Ever smoker at baseline; Panel B: Non-smoker at baseline.

© 2024 Wang K et al. *JAMA Health Forum.*

eFigure 4. Kaplan-Meier curve for all-cause mortality in NAHC group versus usual care only group stratified by Charlson Comorbidity Index (CCI) score

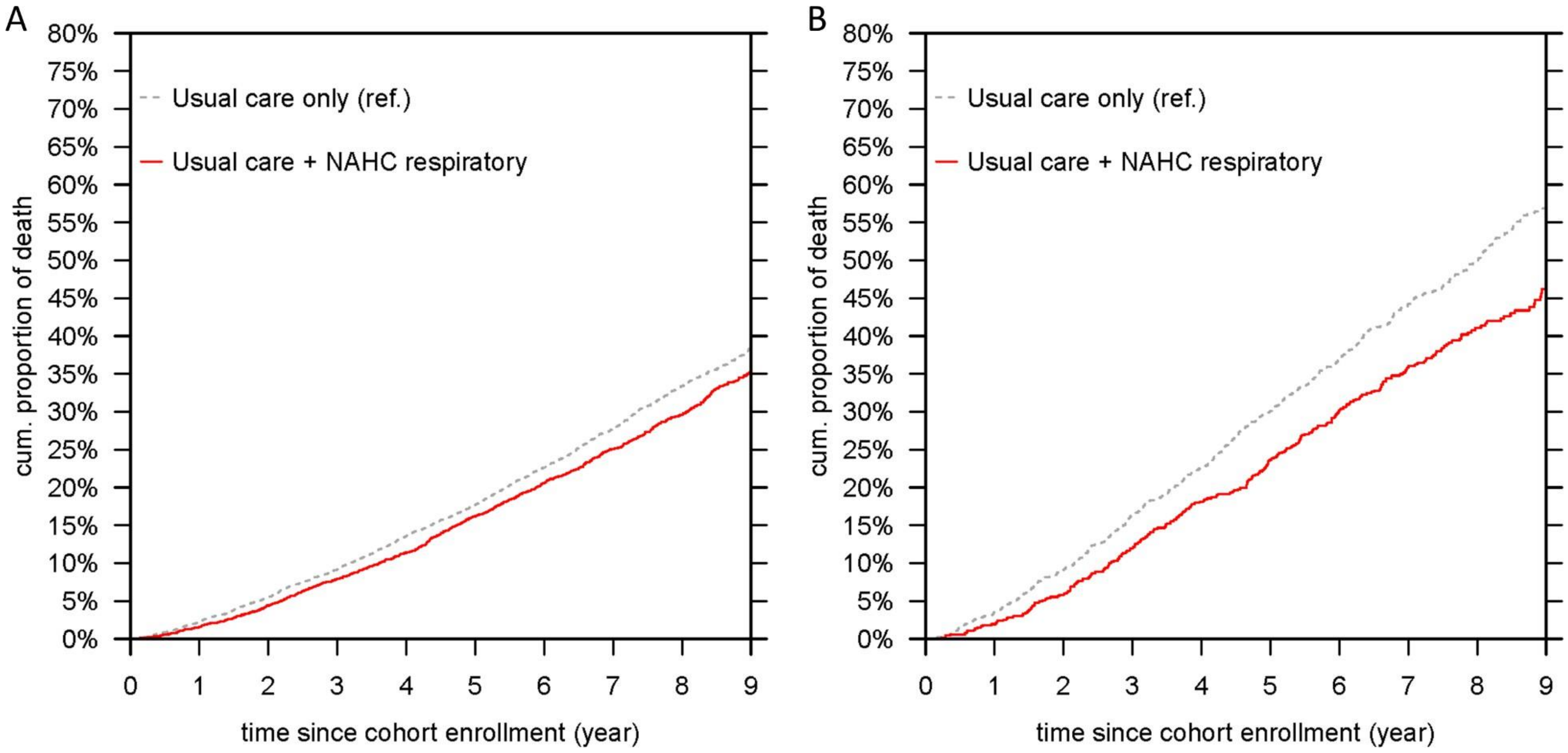

Note: Panel A: CCI score = 0 at baseline; Panel B: CCI score ≥ 1 at baseline. COPD was excluded from the calculation of CCI score here.

© 2024 Wang K et al. *JAMA Health Forum.*

eTable 1. The ICD-10-CM, and ICPC-2 codes used to define the clinical diagnoses

**Diagnoses**	**ICD-10-CM**	**ICPC-2**
Chronic Obstructive Pulmonary Disease (COPD)	J41-J44	R95
Pneumonia	J12-J18	R81
Respiratory failure	J96	-
Lung cancer	C34	R84
Pulmonary heart disease	I27	K82
Pneumothorax	J93	-
Anemia	D50-D64	B80-B82
Polycythemia	D75	-
Depression	F32-F33	P76
Any respiratory disease (for cause of death)	J00-J99	NA
Any cardiovascular disease (for cause of death)	I00-I99	NA
Any cancer (for cause of death)	C00-C97	NA
Hypertension	I10-I13, I15	K86, K87
Asthma	J45	R96
Bronchiectasis	J47	-
Tuberculosis	A15-A19	A70

Note: ICD-10-CM, International Classification of Diseases, 10th revision, Clinical Modification; ICPC-2, International Classification of Primary Care, 2nd edition. ICPC-2 codes were used to identify diagnoses recorded in family medicine datasets. Cause of death only available in ICD-10 code. For diagnoses recorded for the other services, an in-house coding system were used in Hospital Authority Data Collaboration Lab (HADCL) databases, namely term ID. One ICD-10 code (with one letter and three digits) is matched to one or multiple term IDs. The diagnoses used in this study is defined in ICD-10 codes, and selected based on a mapping table provided by HADCL that links ICD-10 codes to term IDs.

© 2024 Wang K et al. *JAMA Health Forum.*

eTable 2. Comparison of baseline characteristics between individuals with and without any missing value at baseline, and between matched and unmatched individuals

	Individuals without vs with missing values			Matched vs unmatched individuals		
	No missing (n=20,437)	With missing (n=2,227)	ASMD	Matched (n=9,048)	Unmatched (n=11,389)	ASMD
Socio-demographics and lifestyle						
Male	82%	80%	0.053	91%	75%	0.372
Age at baseline	71.6	71.4	0.018	69.7	73.1	0.317
Calendar year of cohort enrollment	2011	2011	0.080	2012	2011	0.630
Elderly home residence	3%	7%	0.235	1%	5%	0.190
Reception of public assistance	27%	29%	0.045	26%	28%	0.044
Ever smoker	35%	35%	0.000	45%	28%	0.378
Unknown smoking status	61%	62%	0.020	53%	66%	0.255
Clinical characteristics						
BMI	23.3	23.0	0.059	23.1	23.4	0.081
Asthma	11%	12%	0.031	10%	12%	0.061
Bronchiectasis	2%	2%	0.000	1%	3%	0.118
Tuberculosis	3%	4%	0.063	2%	3%	0.059
Hypertension	51%	33%	0.360	51%	51%	0.000
Charlson comorbidity index	0.5	0.5	0.068	0.4	0.6	0.175
Vaccination against Pneumococcal	22%	17%	0.122	26%	19%	0.179
Duration since COPD diagnosis, yr	0.7	0.4	0.114	0.6	0.7	0.039
Use of long-term oxygen	0%	0%	0.000	0%	0%	0.000
Use of non-invasive ventilation	0%	0%	0.000	0%	1%	0.143
Use of medications						
Use of long-acting beta2 agonists	4%	6%	0.111	2%	4%	0.095
Use of long-acting muscarinic antagonist	1%	2%	0.091	1%	1%	0.000
Use of phosphodiesterase Inhibitors	32%	35%	0.064	27%	35%	0.167
Use of short-acting beta2 agonists	88%	88%	0.000	82%	92%	0.357
Use of short-acting muscarinic antagonist	29%	38%	0.200	24%	32%	0.174
Use of systemic glucocorticoids	59%	63%	0.082	52%	64%	0.250
Healthcare service utilization						
Ever hospitalized in past 3 years	50%	55%	0.100	38%	59%	0.429

© 2024 Wang K et al. *JAMA Health Forum.*

Attendance of any RAMP services for diabetes and hypertension	1%	0%	0.091	2%	1%	0.125
Attendance of day rehabilitation services	2%	3%	0.071	1%	1%	0.000
Attendance of other NAHC services	0%	0%	0.000	1%	0%	0.167
Attendance of smoking cessation services	2%	1%	0.071	5%	0%	0.625

Note: Figures in the table are percentage or mean values. BMI, Body Mass Index; NAHC, Nurse and Allied Health Clinics; RAMP, Risk Assessment and Management Programme

© 2024 Wang K et al. *JAMA Health Forum.*

eTable 3. Sensitivity analysis on risk of mortality among patient alive for at least 2 years after index date

	Cox regression estimation		
	HR	95%CI	P value
**All-cause mortality**			
Usual care group	Ref	-	-
NAHC group	0.86*	(0.79, 0.93)	<0.001
**Cause-specific mortality**			
**COPD**			
Usual care group	Ref	-	-
NAHC group	0.90	(0.70, 1.15)	0.400
**Pneumonia**			
Usual care group	Ref	-	-
NAHC group	0.84*	(0.72, 0.97)	0.016
**Respiratory failure**			
Usual care group	Ref	-	-
NAHC group	1.07	(0.59, 1.94)	0.830
**Lung cancer**			
Usual care group	Ref	-	-
NAHC group	1.06	(0.84, 1.34)	0.630
**Any respiratory condition**			
Usual care group	Ref	-	-
NAHC group	0.85*	(0.75, 0.96)	0.007
**Any cardiovascular condition**			
Usual care group	Ref	-	-
NAHC group	0.76*	(0.59, 0.98)	0.032
**Any cancer**			
Usual care group	Ref	-	-
NAHC group	0.99	(0.84, 1.16)	0.880

*P<0.05. HR, hazard ratio; NAHC, nurse and allied health clinic

© 2024 Wang K et al. *JAMA Health Forum.*

eTable 4. The comparison of the regression results between models with and without adjustment of an additional area variable

	Adjusted by the area variable	Not adjusted by the area variable
	HRs/IRRs (95%CI)	HRs/IRRs (95%CI)
All-cause mortality	0.83 (0.77-0.90)	0.84 (0.78-0.90)
Cause-specific mortality		
COPD	0.90 (0.72-1.13)	0.90 (0.72-1.12)
Pneumonia	0.85 (0.74-0.97)	0.85 (0.74-0.97)
Respiratory failure	1.11 (0.65-1.93)	1.12 (0.65-1.92)
Lung cancer	0.98 (0.80-1.22)	0.99 (0.80-1.22)
Any respiratory condition	0.86 (0.77-0.96)	0.86 (0.77-0.96)
Any cardiovascular condition	0.73 (0.58-0.92)	0.74 (0.59-0.93)
Any cancer	0.94 (0.80-1.09)	0.94 (0.81-1.10)
Subgroups for all-cause mortality		
Age at baseline: <60 years	0.95 (0.67-1.35)	0.97 (0.68-1.37)
Age at baseline: 60-79 years	0.82 (0.74-0.90)	0.82 (0.74-0.90)
Age at baseline: ≥80 years	0.91 (0.80-1.04)	0.91 (0.79-1.04)
Ever smoker: No or unknown	0.91 (0.82-1.01)	0.91 (0.82-1.01)
Ever smoker: Yes	0.75 (0.67-0.84)	0.75 (0.68-0.84)
CCI score: 0	0.88 (0.80-0.96)	0.88 (0.80-0.96)
CCI score: 1+	0.74 (0.64-0.85)	0.74 (0.64-0.85)
Number of admissions		
Emergency room	0.93 (0.87-0.99)	0.92 (0.86-0.98)
Hospitalization	0.94 (0.88-1.00)	0.95 (0.88-1.01)
Emergent hospitalization	0.89 (0.82-0.95)	0.89 (0.83-0.95)
ICU or HDU admission	0.83 (0.51-1.36)	0.84 (0.52-1.35)
Total length of stay		
Hospitalization	0.90 (0.83-0.97)	0.90 (0.84-0.98)
Emergent hospitalization	0.82 (0.75-0.89)	0.82 (0.75-0.88)
ICU or HDU admission	0.80 (0.38-1.68)	0.82 (0.37-1.78)

HR, hazard ratio; IRR, incidence rate ratio; CI, confidence interval

© 2024 Wang K et al. *JAMA Health Forum.*

eTable 5. Risk of diseases incidence in NAHC group versus usual care only group

	Cumulative incidence		Incidence rate per 1,000 person-year			ARR (%)	Cox regression estimation		
	No. of event	Incidence (%)	Rate	Person-year	Median follow-up duration (year)		HR	95%CI	P value
**Pneumonia**									
Usual care group	2229	37.43	65.14	34,221	6.0	-	Ref	-	-
NAHC group	1147	37.08	62.89	18,238	6.2	2.25	0.96	(0.90, 1.03)	0.307
**Respiratory failure**									
Usual care group	843	14.16	22.27	37,848	6.5	-	Ref	-	-
NAHC group	487	15.75	24.30	20,044	6.9	-2.02	1.09	(0.97, 1.22)	0.131
**Lung cancer**									
Usual care group	391	6.57	10.01	39,051	6.7	-	Ref	-	-
NAHC group	203	6.56	9.71	20,915	7.3	0.31	0.97	(0.82, 1.15)	0.702
**Pulmonary heart disease**									
Usual care group	104	1.75	2.66	39,165	6.7	-	Ref	-	-
NAHC group	49	1.58	2.33	21,017	7.3	0.32	0.88	(0.63, 1.23)	0.450
**Pneumothorax**									
Usual care group	155	2.60	3.97	39,003	6.7	-	Ref	-	-
NAHC group	85	2.75	4.07	20,900	7.3	-0.09	1.02	(0.78, 1.33)	0.886
**Anemia**									
Usual care group	574	9.64	15.09	38,047	6.6	-	Ref	-	-
NAHC group	325	10.51	16.00	20,310	7.0	-0.92	1.06	(0.92, 1.21)	0.413
**Polycythemia**									
Usual care group	14	0.24	0.36	39,345	6.7	-	Ref	-	-
NAHC group	8	0.26	0.38	21,072	7.3	-0.02	1.09	(0.45, 2.60)	0.852
**Depression**									
Usual care group	80	1.34	2.04	39,145	6.7	-	Ref	-	-
NAHC group	31	1.00	1.48	20,952	7.3	0.56	0.72	(0.47, 1.09)	0.120

Note: ARR, absolute risk reduction; CI, confidence interval; HR, hazard ratio; NAHC, nurse and allied health clinic.

© 2024 Wang K et al. *JAMA Health Forum.*
