# Supplementary material for: Outcomes and Hospital Service Use Among Patients With COPD in a Nurse- and Allied Health–Led Clinic
Source: JAMA Health Forum. 2024 Jul 5;5(7):e241575. doi: 10.1001/jamahealthforum.2024.1575 (PMC11227079; doi:10.1001/jamahealthforum.2024.1575)
Supplement: Supplement 2. — Data Sharing Statement [file jamahealthforum-e241575-s002.pdf]

Data Sharing Statement

Wang. Outcomes and Hospital Service Use Among Patients With COPD in a Nurse- and Allied Health–Led Clinic. *JAMA Health Forum*. Published July 05, 2024.
doi:10.1001/jamahealthforum.2024.1575

Data
Data available: No

Additional Information
Explanation for why data not available: The data will not be shared due to confidentiality in compliance with the policy of Hospital Authority Data Collaboration Lab (HADCL) released by Hospital Authority of Hong Kong.
